# Charge density mapping demonstrates superiority in catheter ablation of post-surgical atrial tachycardias

**DOI:** 10.3389/fcvm.2024.1453273

**Published:** 2024-10-14

**Authors:** Rita B. Gagyi, Ioan A. Minciuna, Wim Bories, Tamas Szili-Torok

**Affiliations:** ^1^ Department of Internal Medicine, Cardiology Center, University of Szeged, Szeged, Hungary; ^2^ 5th Department of Internal Medicine, Faculty of Medicine, “Iuliu Hațieganu” University of Medicine and Pharmacy, Cluj-Napoca, Romania; ^3^ Acutus Medical Inc., Zaventem, Belgium

**Keywords:** atrial tachycardia, cardiac mapping, global chamber mapping, catheter ablation, mapping

## Abstract

**Introduction:**

Atrial tachycardia (AT) frequently occurs after cardiac surgery or surgical ablation procedures. The novel charge density-based mapping system (CDM) provides global chamber mapping and can detect crucial pathways of conduction; therefore, it has potential added value in catheter ablation (CA) of post-surgical ATs. We aimed to test the hypothesis that CDM-guided CA procedures are safe, feasible, and may improve outcome compared to conventional sequential 3D mapping (CARTO)-based CA.

**Methods and results:**

Consecutive patients undergoing CA for post-surgical AT guided by CDM or CARTO were enrolled. Procedural safety and efficiency were analyzed. Acute success, one-year outcome was assessed. A total of 35 patients (mean age 60.8 ± 10.6 years, 42.9% female) underwent CA of AT using CDM (*n* = 20) and CARTO (*n* = 15). A total of 61 ATs were mapped (35 in CDM and 26 in CARTO group). Four patients had focal ATs, 22 macro re-entrant, and 8 patients had ATs with both mechanisms. No differences were found in procedural complication (CDM 3 vs. CARTO 1 patient, *p* = 0.61). There were no differences in procedure duration (185.9 vs. 147.9 min, *p* = 0.09), fluoroscopy dose (165.0 vs. 155.0 mGy, *p* = 0.31), RF application number (28.0 vs. 18.0, *p* = 0.17) or duration (1,251.5 vs. 1,060.0 s, *p* = 0.54). Acute success was 95.0% in CDM and 73.3% in CARTO group (*p* = 0.14). Cumulative AT recurrence rates were lower in CDM group compared to CARTO group (10.0% vs. 46.7%, *p* = 0.02).

**Conclusions:**

The CDM system is feasible. Our data suggest that patients treated with CDM-guided CA developed fewer AT recurrences as compared to CARTO-guided procedures.

## Graphical Abstract

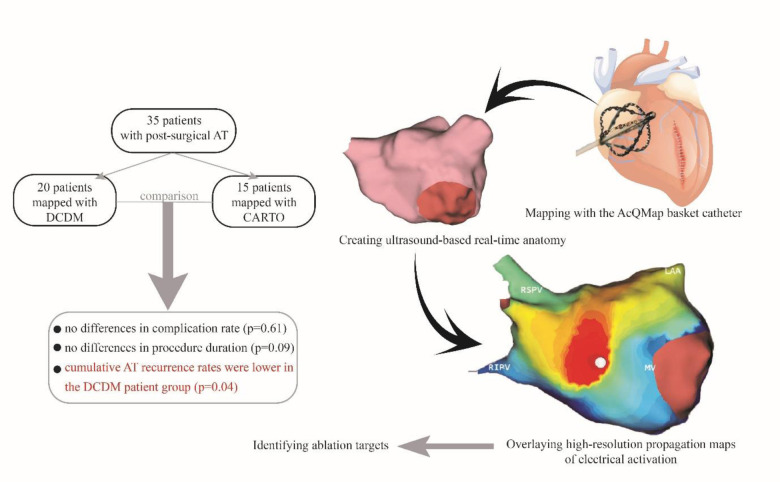

## Introduction

Atrial tachyarrhythmia (AT) is frequently encountered in patients after surgical ablation or cardiac surgery, and is associated with impaired quality of life, substantial morbidity—including heart failure– and mortality (1–4). Catheter ablation (CA) is a widely accepted treatment strategy for post-surgical ATs. However, mapping and ablation of these arrhythmias is often technically challenging due to altered anatomy, complex atrial substrate, and instability of the AT with cycle length (CL) variations. Consequently, with current sequential mapping techniques these procedures are time-consuming with higher recurrence rates and more repeat ablations compared to ATs in structurally normal heart (5).

The AcQMap mapping system (Acutus Medical, Carlsbad, CA) is a novel charge density based mapping (CDM) system capable of simultaneous ultrasound based anatomy reconstruction and global chamber mapping offering high-resolution propagation maps of the whole atrium (6). Importantly, it can identify the mechanism by using single beat mapping, without the confounding effects of far-field signals and without the influence of CL variations or changes in activation patterns (7). Recent studies have reported the feasibility, safety and benefit of this mapping technology over conventional sequential mapping systems for other arrhythmias (8, 9). However, the use of the CDM system has not been studied in post-surgical ATs.

The aim of the study was to assess (1) the safety and feasibility of the CDM system in CA procedures of patients with post-surgical AT and (2) one-year outcome compared to conventional sequential 3D mapping based CA.

## Material and methods

### Study population

The study population consisted of consecutive adult patients with post-surgical AT documented on ECG recordings or Holter monitoring who underwent CA using either the CDM or CARTO 3D mapping systems during the same period of time (2018–2022). All patients were excluded with the presence of intracardiac baffles (regardless of containing prosthetic or human tissue).

### Consent and ethics

The institutional medical ethics committee (Erasmus MC Medical Ethics Review Committee—MERC) approved the data collection for this study and concluded that it did not fall under the Medical Research Involving Human Subjects Act (SERCA-2, MEC-2021-0299). The study was performed according to the principles of the Declaration of Helsinki.

### Primary hypothesis and study design

The primary hypothesis of this study was that the CDM system is equally safe and feasible as compared with the CARTO mapping system. Further, we investigated the hypothesis that CDM might offer advantages as compared to CARTO mapping system in one-year outcome for post-surgical AT ablation. The primary endpoints of this study were safety characterized by procedure related complications and efficiency characterized by procedure duration, fluoroscopy use, radiofrequency application number, duration and acute procedural success. The secondary endpoint was procedural efficacy characterized by AT (and/or atrial fibrillation) recurrence during a 12-months follow-up and number of redo procedures.

### Definitions

Major complications were defined as any procedure-related adverse event, which were life threatening, requiring significant surgical intervention and prolonged hospital stay or resulted in death. Major complications comprise permanent second- or third-degree atrioventricular block, cardiac tamponade, hemorrhagic shock, stroke, and procedure-related death. Minor complications were defined as procedure-related adverse events, which resulted in minimal transient impairment of a body function or damage to a body structure, or which did not require any intervention or further therapy, such as local hematoma in the puncture site, temporary bundle branch block or AV block, pericardial effusion not requiring intervention. We considered as access site complications: bleeding complications or local vascular wall damage, which required surgical intervention or intervention by radiologist, prolonged hospitalization and/or hemoglobin drop of >1.8 mmol/L (2.9 g/dl). Total procedure time was defined as the time passed from first venous puncture until the removal of sheaths. Acute success was defined as conversion to sinus rhythm during radiofrequency application and/or non-inducibility of the treated arrhythmia. Non-inducibility was defined as inability to induce any arrhythmia with or without administration of isoproterenol and with programmed atrial stimulation up to 3 extra stimuli and incremental burst pacing not below 200 ms. Recurrence was defined as any documented AT regardless of its duration.

### Data collection

Baseline demographic, clinical characteristics and procedural data from patients were collected from our prospective database using the electronic health records (HiX version 6.1) and analyzed in accordance with the hospital institutional review board policies. We collected safety data, including acute intra-procedural and post-procedural adverse events, efficiency data such as acute procedural success, efficacy data such as AT recurrence, and number of redo procedures. The following demographic and procedural data were collected: age, sex, weight, height, BMI, procedure duration time, fluoroscopy dose, number of applications, application duration, and rhythm at the end of procedure. Further, we collected and analyzed clinical data, such as surgical history, echocardiography data (left atrial dimension, left ventricular ejection fraction, left atrial volume index), comorbidities, and antiarrhythmic medication. Mapping data were collected from the AcQMap workstation.

### Dipole charge density and AT mapping

**Figure 1:**
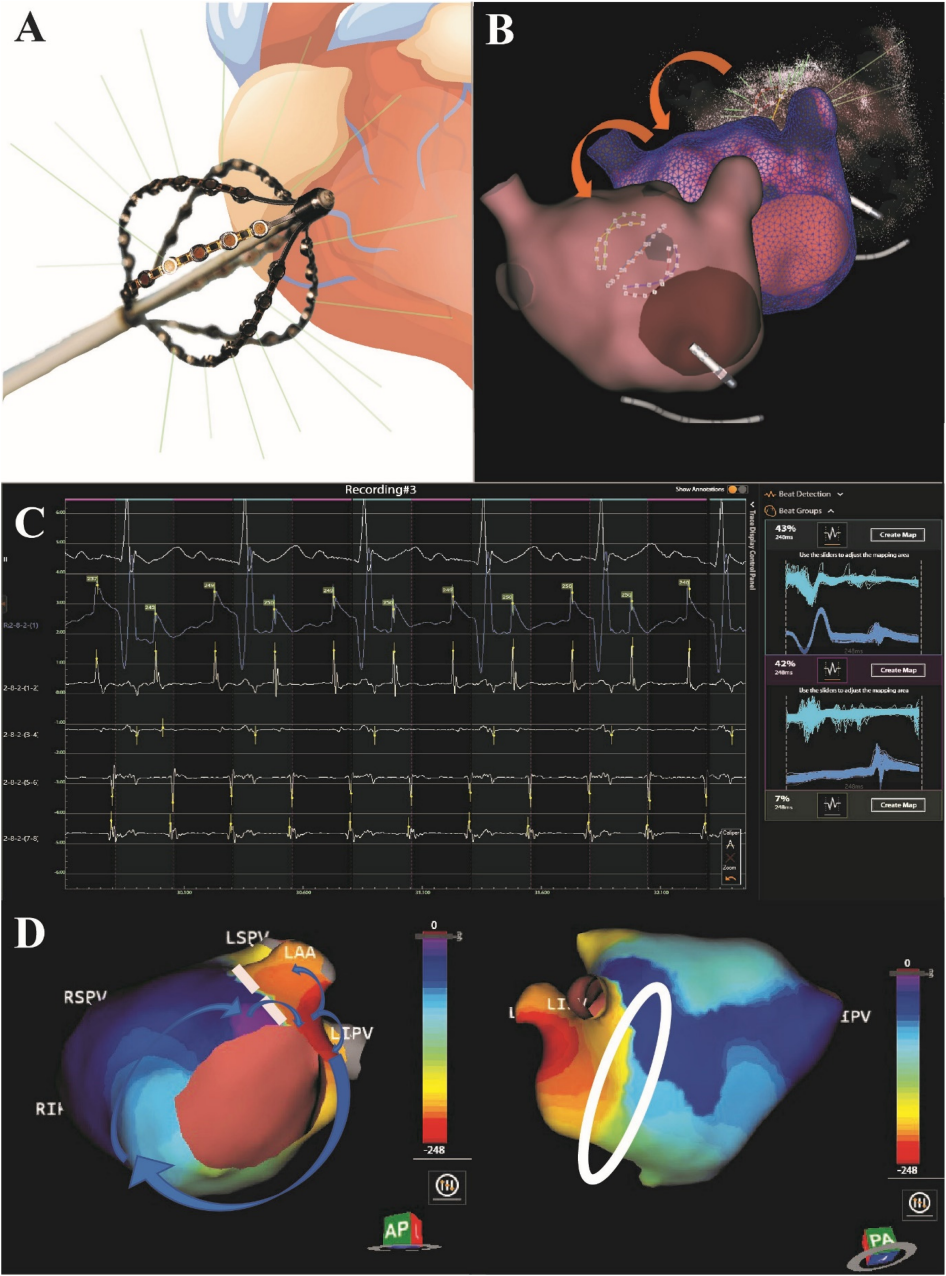
Summary of the CDM workflow. The 48-pole basket mapping catheter is shown in panel **(A)**. After introducing the basket catheter in the chamber of interest, first an ultrasound-based real time anatomy is reconstructed **(B)** which corresponds to the end-diastolic size and shape of the atrium. Unipolar intracardiac potentials are sensed from the biopotential electrodes of the basket catheter and are processed by an inverse solution to derive the dipolar charge sources at the endocardial surface. The SuperMap feature performs automatic beat grouping based on uni-, and bipolar reference signals that share similar morphology. SuperMap in the left atrium shows a tachycardia with 248 ms CL and 2-to-1 conduction to the ventricle **(C)**. The waves of electrical activation are displayed across the 3D anatomy through time as high-resolution propagation history maps **(D)**. Panel D shows an example of a patient treated with post-maze atrial tachycardia. During the mapping, an atypical flutter was observed in the left atrium through the surgical lines. A second, double-loop re-entry tachycardia was mapped in the right atrium. Blue arrows show the direction of the propagation on the LA. White circle highlights the bunching of isochrones showing a delay, which indicates slow conduction.

The CDM noncontact mapping system is a charge density based mapping technology that combines ultrasound-based 3D endocardial anatomy reconstructions with high-resolution propagation history maps of electrical activation and allows visualization of global atrial activation. The 48-pole basket mapping catheter (AcQMap catheter, Acutus Medical, Carlsbad, CA) has six splines, each spline incorporating eight biopotential electrodes and eight ultrasound transducers. The ultrasound-generated 3D endocardial chamber surface reconstruction corresponds to the end-diastolic size and shape of the atrium. Unipolar intracardiac potentials are sensed from the biopotential electrodes of the basket catheter and are processed by an inverse solution to derive the dipolar charge sources at the endocardial surface. The waves of activation are displayed across the 3D anatomy through time as high-resolution propagation history maps. The noncontact modality of the CDM system allows for two modalities of mapping: single position mapping (SPM) and aggregated multiposition noncontact mapping (SuperMap). SPM can be applied as single-beat analysis to map short non-sustained ATs or PACs. While SuperMap enables mapping of both non-sustained and sustained repetitive atrial rhythms. A summary of the CDM workflow is illustrated on Figure 1.

### Catheter ablation

All procedures were performed with the use of the robotic magnetic navigation system (Niobe, Stereotaxis Inc., St. Louis, MO, USA). After the introduction of catheters, if the patients were in sinus rhythm, attempts of AT induction were made including atrial programmed extrastimulation and burst atrial pacing. If this was unsuccessful or when AT did not occur spontaneously, isoproterenol was administered. Entrainment mapping was performed at the discretion of the operator. In cases when CARTO (version 6) was used, we made electro-anatomic reconstructions and analyzed activation maps. Maps of the right and left atrium were made using the Navistar RMT ThermoCool ablation catheter (Biosense Webster, Irvine, CA). Activation mapping was performed using PentaRay catheter (Biosense Webster Inc., Irvine, USA) and Lasso (Biosense Webster Inc., Irvine, USA) mapping catheters. The PentaRay mapping catheter was used in 4 (27%) procedures and the Lasso catheter was used in 11 (73%) procedures (Table 1). When CDM was used, after reconstructing the endocardial anatomical surface, we overlaid high-resolution charge density base maps of electrical activation using the CDM system. CD maps were performed manually. We interpreted propagation history maps, identified atrial activation patterns, and performed targeted ablation using the MagnoFlush (Medfact, Germany) ablation catheter. When using CARTO, after identification of the chamber of origin, mechanism and location of the substrate, ablation was performed using the NaviStar RMT ablation catheter. The following settings were used in both study groups: 45–50 W (posterior wall—anterior wall, respectively), 17 ml/min flow rate, maximum 43℃. Intravenous heparin was administered for anticoagulation, guided by activated clotting time (>350 s for LA and >300 s for RA when the CDM was used, in patients mapped with CARTO the target was 300 s).

**Table 1:** Characteristics of the CARTO group.

Patient				Post-surgical AT	Radiofrequency ablation	Mapping catheter
No.	Sex	Age	Surgical procedure	Site	Ablation lines	
1	F	42	PAPVR repair	RA scar, septum, SVC	Bicaval line, line from SVC to TVA	PentaRay
2	F	42	VSD closure	CTI	CTI line	Lasso
3	M	70	Vats-MAZE, CABG, AD	Perimitral	Anterior line	PentaRay
4	M	61	Bidirectional Glenn, ToF	CTI	CTI line	PentaRay
5	F	53	ToF, PVR, VSD closure	–	–	Lasso
6	F	50	ToF, PVR, TVR	High RA	CTI line	Lasso
7	M	56	Vats-MAZE	Around VCI, LA roof	Bicaval line, roof line	PentaRay
8	M	56	ASD-II, PAPVR repair	CTI	CTI line	Lasso
9	M	59	ASD-II, MVP	LA	–	Lasso
10	M	72	VSD closure	CTI, posterior scar, CS ostium	CTI line, line between scar, SVC and IVC	Lasso
11	F	70	Septal myectomy	Septum, mid IAS	Septal line, mitral isthmus line, posterior wall isolation (Box) and CS ablation	Lasso
12	M	76	Vats-MAZE	Septum, CTI, LA	Trigonum line, RA supero-posterior	Lasso
13	M	57	ToF	CTI, atriotomy scar	Bicaval line, CTI line, line between VCS and atriotomy	Lasso
14	F	64	PAPVR repair	RA lateral, CTI	CTI to TVA	Lasso
15	F	63	TAPVR repair, ASD-II	Septum, LA roof, LSPV	–	Lasso

PAPVR, partial anomalous pulmonary venous return; VSD, ventricular septal defect; CABG, coronary artery bypass grafting; AD, type A aortic dissection with hemi-arc replacement; TVR, tricuspid valve repair; ASD, atrial septal defect; MVP, mitral valve repair; ToF, Tetralogy of Fallot repair; TAPVR, total anomalous pulmonary venous return; RA, right atrium; LA, left atrium; SVC, superior vena cava; IVC, inferior vena cava; CS, coronary sinus; RSPV, right superior pulmonary vein; LSPV, left superior pulmonary vein; TVA, tricuspid valve annulus.

### Follow-up

After every procedure, patients were continuously monitored by 24-h telemetry. Pre-discharge echocardiography and regular access site checks were performed in all patients in order to screen post-procedural complications.

Patients were followed up with a routine 3-, 6- and 12-month hospital visit. During the follow-up visits 24-h (3 and 6 months) and 7-day Holter recordings (at 12 months) were analyzed for documentation of recurrent arrhythmias. Patients with recurrences were considered for repeated CA procedure or were identified as ablation failures.

### Statistical analysis

All analyses were carried out using the SPSS software version 28.0.1 (IBM SPSS Inc., Chicago, IL). Mean and standard deviation (SD) were calculated for normally distributed continuous variables. Median and interquartile range (IQR) were computed for continuous variables with non-normal distribution. Normality of distribution was assessed with skewness. Descriptive statistics for categorical data were expressed in absolute numbers and percentages. Statistical significance was defined as *p* < 0.05 (two-tailed). Data showing normal distribution were compared using independent samples *t*-test, while non-normally distributed variables were analyzed using the Mann-Whitney *U*-test. The chi-square test was used to compare categorical variables between groups (Pearson Chi-Square or Fisher's Exact Test).

## Results

### Study population

Thirty-five consecutive patients (mean age 60.8 ± 10.6 years, 42.9% female) scheduled for post-surgical AT ablation were included. Baseline characteristics of the study population are summarized in Table 2. The CDM group consisted of 20 patients (10 patients with previous surgical ablation, 10 patients with previous cardiac surgery), described in Table 3. The CARTO group consisted of 15 patients (3 patients with previous surgical ablation and 12 with previous cardiac surgery), described in Table 1. In the overall patient group, 13 patients underwent previous surgical ablation and 22 had at least one previous cardiac surgery.

**Table 2:** Baseline characteristics.

	All patients *N* = 35	CDM *n* = 20	CARTO *n* = 15	*P*-value
Mean age, years (±SD)	60.8 ± 10.6	61.8 ± 11.0	59.4 ± 10.2	0.51
Female (%)	15 (42.9%)	8 (40.0%)	7 (46.7%)	0.74
BMI (±SD)	27.3 ± 4.9	26.8 ± 3.7	27.8 ± 6.3	0.56
Weight (kg)	82.9 ± 15.0	84.1 ± 15.4	81.5 ± 14.4	0.62
Height (cm)	174.9 ± 11.8	176.9 ± 11.7	172.1 ± 11.6	0.22
Heart failure (%)	7 (20.0%)	4 (20.0%)	3 (20.0%)	1.00
Ischemic heart disease	3 (8.6%)	2 (10.0%)	1 (6.7%)	1.00
Congenital heart disease	14 (40.0%)	4 (20.0%)	10 (66.7%)	0.01
Lung transplant	1 (2.9%)	1 (5.0%)	0 (0.0%)	1.00
Hypertension	16 (45.7%)	11 (55.0%)	5 (33.3%)	0.30
Cardiomyopathy	3 (8.6%)	2 (10.0%)	1 (6.7%)	1.00
Diabetes	4 (11.4%)	3 (15.0%)	1 (6.7%)	0.61
CVA or TIA	5 (14.3%)	4 (20.0%)	1 (16.7%)	0.36
OSAS	0 (0.0%)	(0.0%)	0 (0.0%)	N/A
Echocardiography data				
Preoperative LVEF (%)	52.1 ± 5.8	52.0 ± 6.1	53.1 ± 5.1	0.60
LA diameter (mm)	49.1 ± 10.8	45.9 ± 4.7	52.4 ± 14.2	0.26
LAVI (ml/m^2^)	41.7 ± 9.5	42.5 ± 9.4	33.0 ± 1.4	0.20
TAPSE	17.5 ± 4.0	18.5 ± 4.7	16.6 ± 2.4	0.27

CDM, charge density mapping; SD, standard deviation; BMI, body mass index; CVA, cerebral vascular attack; TIA, transient ischemic attack; OSAS, obstructive sleep apnea syndrome; LVEF, left ventricular ejection fraction; LA, left atrium; LAVI, left atrial volume index; TAPSE, tricuspid annular plane systolic excursion; N/A, not applicable.

**Table 3:** Characteristics of the CDM group.

Patient				Post-surgical AT	Radiofrequency ablation
No.	Sex	Age	Surgical procedure	Site	Additional lines
1	F	69	Vats-MAZE	Low septum, high RA	–
2	M	67	Vats-MAZE	LSPV, LIPV, perimitral	Anterior line, RSPV to mitral annulus
3	M	63	AVR, MVR, Cox-MAZE IV	Lateral edge of TA	–
4	M	74	Vats-MAZE	Perimitral	Anterior line
5	M	50	Vats-MAZE	Anterior and posterior mitral line, septum	Line over the fossa ovalis from VCS to VCI
6	M	51	ToF, PVR, PVI, ASD-II	CTI	–
7	M	73	MVR, MAZE, LAAA	Posterior under MV, anterior wall	RSPV to mitral annulus
8	M	57	Vats-MAZE	LIPV, CTI, ventricular ridge	Inferior line, posterior box lesion
9	F	67	Cox-MAZE IV, MVR, TVP	LA roof, septum	–
10	M	75	CABG, MVR	Lateral RA wall	–
11	M	57	CAGB	Under LIPV	Anterior line, mitral line, roof line
12	F	54	ToF, PVR	Low posterior LA	–
13	F	45	Bentall	Waterston scar, RAA, high lateral	–
14	F	68	LTx	LA roof	–
15	M	76	MVR	CTI	CTI line
16	F	48	VSD repair, MVR	CTI, posterior to IVC, Waterston scar	CTI line
17	F	40	Cone repair, ASD-II, bidirectional Glenn	Lateral scar, IVC and TV	–
18	M	63	MVR, TVP, CABG	Poster-superior RA, Waterston scar	CTI line
19	F	76	MVR	RA lateral wall	–
20	M	63	AVR, MAZE	Near RIPV, anterior septal	Mitral isthmus line and posterior atrial line

AVR, aortic valve replacement; MVR, mitral valve replacement; LAAA, left atrial appendage amputation; ToF, Tetralogy of Fallot repair; PVR, pulmonal valve replacement; ASD, atrial septal defect repair; TVR, tricuspid valve repair; CABG, coronary artery bypass grafting; LTx, lung transplant; VSD, ventricular septal defect; TA, tricuspid annulus; MV, mitral valve; RA, right atrium; RAA, right atrial appendage; TV, tricuspid valve; RSPV, right superior pulmonary vein; VCS, vena cava superior; VCI, vena cava inferior.

### Safety data

Three patients in the CDM group presented post-procedural complications: one minor complication (access site hematoma) and two major complications (cerebrovascular attack and haemothorax). The patient suffering a CVA was considered a mild case and was discharged after 2 days (National Institutes of Health Stroke Score = 1). One patient in the CARTO group presented a major post-procedural complication (intraoral bleeding due to injury during intubation) which was not a result of cardiac mapping. There was no difference in complication rates between the two groups (*p* = 0.61).

### Atrial tachycardia characteristics

**Figure 2:**
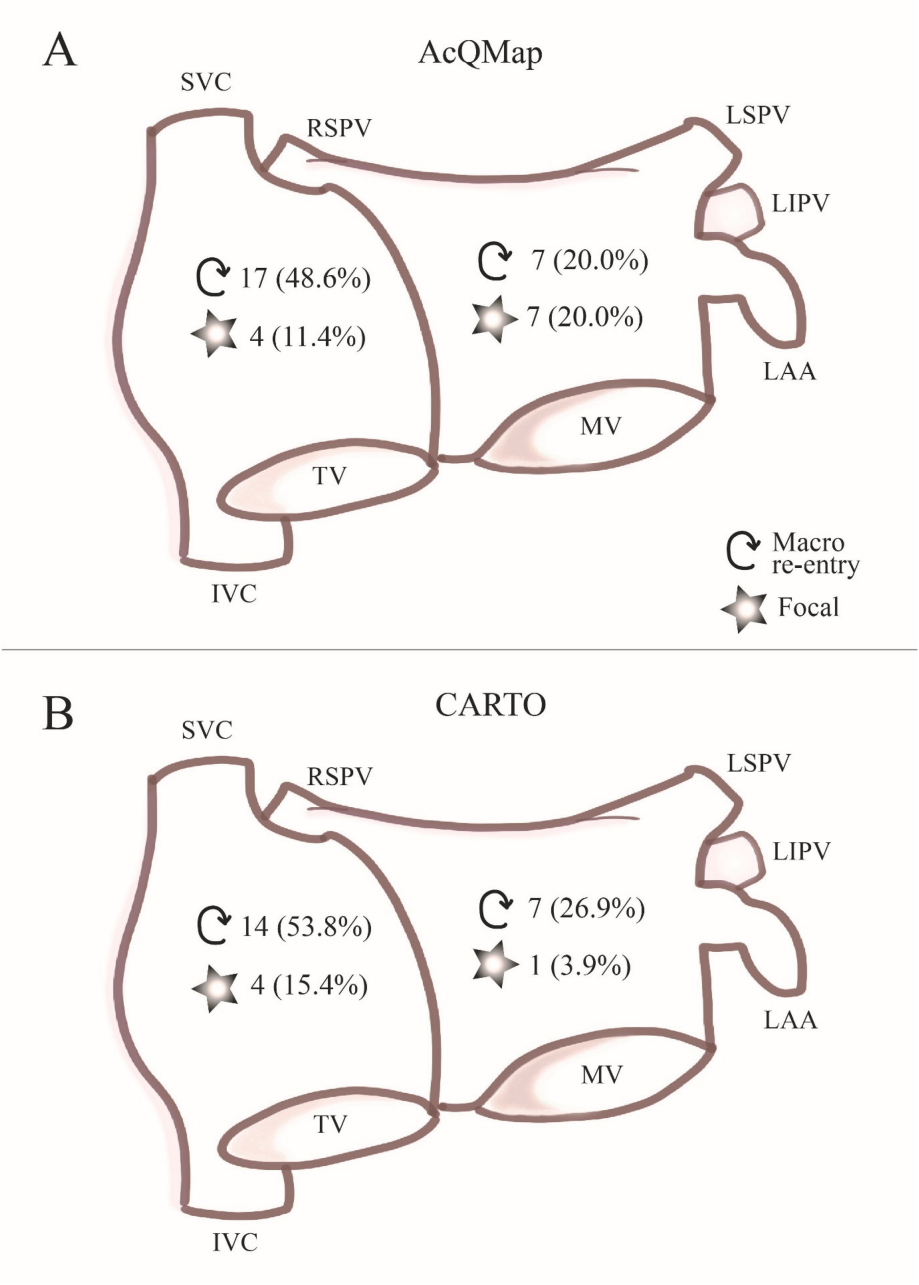
AT characteristics. In panel **(A)** chamber of origin and mechanism of ATs mapped with the CDM system is summarized. In panel **(B)**, chamber of origin and mechanism of ATs mapped with the CARTO mapping system is summarized.

We mapped a total number of 61 ATs in 35 patients (35 in the CDM group and 26 in the CARTO group, 1.6 ± 0.7 ATs per patient, (1–3). Sixteen out of 61 ATs showed focal origin (11 in CDM and 5 in CARTO group) and 45 showed macro re-entry mechanism (24 in CDM group and 21 in CARTO group) and there was no difference between the patient groups (*p* = 0.72). Thirty-nine ATs were mapped in the right atrium (RA) (21 in CDM (60%) and 18 in CARTO (69%) group) 22 in the left atrium (LA) (14 in CDM (40%) and 8 in CARTO (31%) group) (Figure 2). Four patients had focal ATs, 22 patients had macro re-entrant ATs, and 8 patients had multiple ATs with both mechanisms. In one patient AT mechanism could not be identified. There were no differences in AT mechanism between the patient groups (*p* = 0.72). In the CDM group, 3 patients had ATs in both atria (15.0%), in the CARTO group 4 patients (26.7%) had ATs both in right and left atrium (*p* = 0.59). In the CDM guided procedures, single-position mapping was performed exclusively in 11 patients, aggregated multi-position mapping was performed exclusively in 5 patients, and both SPM and SuperMap were performed in 4 patients.

### Procedural efficiency and outcome

There were no differences in procedure duration (185.9 vs. 147.9 min, *p* = 0.09), fluoroscopy dose (165.0 IQR 121.0–254.0 vs. 155.0 IQR 15.0–286.0 mGy, *p* = 0.31), RF application number (28.0 IQR 16.5–60.5 vs. 18.0 IQR 6.0–39.0, *p* = 0.17) or duration (1,251.5 IQR 690.5–1,817.5 vs. 1,060.0 IQR 450.0–2,073.0 s, *p* = 0.54) between the two groups. Subgroup analysis for patients with previous cardiac surgery is summarized in Table 4. Acute success rate of AT ablation was 95.0% in the CDM group and 73.3% in the CARTO group (*p* = 0.14), and did not differ between groups when comparing data of patients with previous cardiac surgery (90.0% vs. 66.7%, *p* = 0.32). AT termination during RF application was observed in 17 patients in the CDM group and in 10 patients in the CARTO group (*p* = 0.24). Electrical cardioversion (ECV) was performed in two patients in the CDM group and seven patients in the CARTO group (*p* = 0.02) either for AT or AF. In one patient in the CDM group, the arrhythmia was terminated during entrainment mapping. In the subgroup analysis for patients with previous cardiac surgery, we found that there were no differences in AT termination and ECVs performed between the two mapping systems (termination 8 vs. 7, *p* = 0.38 and ECV 1 vs. 6, *p* = 0.07).

**Table 4:** Subgroup analysis for patients with previous cardiac surgery.

	Patients with previous cardiac surgery *N* = 22		*P*-value
	CDM group *N* = 10	CARTO group^a^ *N* = 12	
Procedural data			
Procedure duration (min)	186.1 ± 73.6	146.5 ± 54.0	0.16
Fluoroscopy dose (mGy)	152.0 (IQR 85.0–266.3)	66.5 (IQR 15.0–250.8)	0.22
Number of RF applications	22.0 (IQR 14.0–75.8)	18.0 (IQR 7.0–44.0)	0.45
Application duration (sec)	965.0 (IQR 607.0–2,897.5)	1,149.0 (IQR 462.3–1,869.0)	0.67
Termination during RF application (%)	8 (80.0%)	7 (58.3%)	0.38
Tachycardia characteristics			
Number of ATs mapped	15	19	0.50
Focal mechanism/patient (%)	1 (10.0%)	1 (8.3%)	0.99
Re-entry mechanism/patient (%)	8 (80.0%)	9 (75.0%)	
Both focal and re-entry mechanism/patient (%)	1 (10.0%)	1 (8.3%)	
AT in the RA (%)	7 (70.0%)	6 (50.0%)	0.36
AT in the LA (%)	3 (30.0%)	3 (25.0%)	
AT in both LA and RA (%)	0 (0.0%)	2 (16.7%)	
Outcome data			
Acute success (%)	9 (90.0%)	8 (66.7%)	0.32
Cumulative AT recurrence (%)	0 (0.0%)	6 (50.0%)	0.01
Freedom from all arrhythmias (%)	10 (100.0%)	6 (50.0%)	0.01

IQR, interquartile range; RF, radiofrequency; AT, atrial tachycardia; RA, right atrium; LA, left atrium.

^a^ In one patient AT was not mappable with the CARTO system due to technical issues; empiric ablation was performed.

### Tachycardia site and additional radiofrequency ablation lines

A more detailed description of the sites of the mapped post-surgical ATs and additional ablation lines are shown in Table 3 for CDM group and Table 1 for the CARTO patient group.

### Follow-up

At the 3-months follow-up visit none of the patients had recurrences in the CDM group and 4 patients had recurrences in the CARTO group (0.0% vs. 26.7%, *p* = 0.02). At the 6-months follow-up visit one patient presented with recurrence in the CDM group, and 5 patients had recurrences in the CARTO group (5.0% vs. 33.3%, *p* = 0.06). During the 12-months follow-up visit one additional patient presented with AT recurrence in the CDM group, and 5 patients presented with recurrence in the CARTO group (5.0% vs. 33.3%, *p* = 0.06). At the end of the follow-up period, we documented a cumulative AT recurrence rate of 10.0% (2 out of 20 patients) in the CDM group and 46.7% (7 out of 15 patients) in CARTO group (*p* = 0.02). Two patients presented with atrial fibrillation (AF) recurrence in CDM group (2 out of 20, 10.0%), and 3 patients (20.0%) in CARTO group (*p* = 0.63). Freedom from all arrhythmias was documented in 14 (out of 20) patients in the CDM group and 7 (out of 15) patients in CARTO group (*p* = 0.02) at the end of the follow-up period. Subgroup analysis for patients with previous cardiac surgery is summarized in Table 4. Nine patients continued with antiarrhythmic medication in the CDM group (45.0%) and 12 patients (80.0%) in the CARTO group (*p* = 0.04). ECV was performed in two patients in the CDM group during the 12-months follow-up period, and in two patients in the CARTO group (*p* = 1.00). One patient in the CDM group required a redo procedure and two patients (13.3%) in the CARTO group underwent a redo procedure within 12-months (*p* = 0.56).

## Discussion

To the best of our knowledge, this is the first study aiming to examine the safety and feasibility of the dipole charge density mapping system (CDM) in comparison with the conventional sequential 3D mapping (CARTO) system in the setting of post-surgical AT ablation. The main finding of our study is that that the CDM system is feasible, and might offer advantages compared to the CARTO mapping system in one-year outcome of CA procedures for post-surgical ATs. These findings contribute in several ways to our understanding of post-surgical AT mechanisms, and might provide a basis for future CA approaches for these complex arrhythmias.

### Post-surgical AT mapping and ablation

According to the current literature, depending on the type of previous cardiac surgery, a range of 10%–37% of patients develop post-surgical ATs (1, 10–13). These patients have altered atrial myocardium due to a combination of longstanding residual pressure, volume overload, and surgical scarring which results in higher incidence of ATs (14). Changes in atrial refractoriness, sinus node dysfunction and the presence of slow conduction zones leads to more than one AT circuit in many patients (15). The administration of antiarrhythmic medication and atrial pacing did not demonstrate effectiveness in post-surgical AT treatment (4, 15). Studies show, however, that radiofrequency CA is offering a long-term solution and electroanatomic mapping systems may improve the outcome of such ablation procedures (14, 16–18). Electrophysiology (EP) studies for patients with post-surgical ATs are often challenging due to complex anatomy, varying CL and unstable nature of ATs. In the early 90's the most popular approach to treat post-surgical ATs was targeting slow conduction zones identified by electrogram timing and fractionation (19). With the improvement of technology and catheter design, using entrainment techniques to identify critical isthmuses became more and more widespread, too (20). In a study conducted by Kalman et al. in 1996, using entrainment to ablate the critical isthmus, the authors report an acute success rate of 83%, and documented a recurrence rate of 50% (9 out of 18) (20). In the last decades, it became clearer that while entrainment mapping is a cornerstone in EP studies, in patients with post-surgical ATs it frequently changes or terminates the arrhythmia (14). Further, entrainment mapping may have limited efficacy in patients with extensive atrial scars due to low-amplitude or absent atrial potentials and the difficulty to capture the atrial myocardium. Over the years, several attempts have been made to accurately map post-surgical or incisional tachycardias using different mapping systems, such as the Rhythmia mapping system (21, 22). Although the newer systems offer a high-resolution map and reliable identification of surgical scar, they require stable CL during mapping. Therefore, they might face difficulties when multiple and unstable ATs are present with rapidly changing CL. Further, authors suggest that the mini-basket catheter utilized by the Rhythmia system can be challenging to manipulate because of its shape and might result in termination of the arrhythmia during the mapping process (22). Standard 3D electroanatomic mapping systems, such as the CARTO mapping system and new generation NavX provided a promising alternative in the treatment of these challenging arrhythmias. However, they faced similar limitations: unstable nature of post-surgical ATs with varying CL and frequent termination during mapping. When failing to induce the ATs or when the AT is unstable with variable CL, mapping is often performed during atrial pacing, by the detection of slow conduction zones. It is important to mention that functional blockade does not always correlate with anatomical barriers; therefore, additionally to performing lines of conduction block, visualizing activation patters in the whole atrium would be an optimal approach in these patients. In a recent publication by Shen et al. (23) a part of the mapping system module in CARTO (ripple mapping) was utilized proving to improve diagnostic accuracy and ablation outcome compared with conventional local activation time mapping and entrainment mapping, especially in complex left atrial AT (after MAZE procedure). In their study of 34 patients, an acute success of 94.1% and a long-term success of 79.4% was documented. This approach requires continuous manual adjustment of voltage threshold in order to localize and display the electrophysiological isthmus. Although ripple mapping graphically displays the complexity of electrograms, it does not facilitate the issue of interpretation with regard to far-field signals and spatial resolution (24). More recent studies using conventional mapping techniques to ablate post-surgical ATs show similar results as our CARTO patient group (18). Anne et al. document a 29% recurrence rate at 24-months and 13% redo ablation rate in a study of 45 patients with post-surgical ATs (5). According to our results, the CARTO study group had a lower incidence of acute arrhythmia termination by ablation, and an increase in late AT recurrence. This could be explained by failure of accurate mapping, but late recurrence might also be influenced by RF lesion durability, or the emergence of new arrhythmia circuits from possible pro-arrhythmic ablation lesions. Three-dimensional visualization of surgical scars and anatomical barriers seemed to result in better acute outcomes but long-term results still remain suboptimal. The inability of accurately map complex activation patterns due to the above mentioned limitations might be a reason for these suboptimal results.

### Dipole charge density mapping

The CDM system uses ultrasound to reconstruct and delineate the anatomy in detail, which aids in accurate navigation and targeting of the arrhythmia. The acquisition of the real-time anatomy takes up to 2–3 min, after which consecutive activation history maps can be performed on a beat-to-beat basis (25). Although reports of this technique in patients with complex anatomy was limited before (7), we demonstrated the beneficial use of intra-operative ultrasound-guided imaging in a substantial number of patients with complex CHD and acquired heart defects, who underwent previous cardiac surgery. The CDM system facilitates most of the above-mentioned limitations with single position and SuperMap mapping functions. While the first permits complete atrial mapping of every beat, the latter might render entrainment obsolete due to its highly accurate mapping capabilities. The major advantage of single position noncontact simultaneous global chamber mapping is, that it is not dependent of a reference. On the other hand, for SuperMap mapping a reference is necessary. Stability of the reference is less important, as the algorithm automatically assigns beats to the sequence of the reference signal. This mapping mode is more similar to the CARTOFINDER system (26).

The safety and feasibility of the CDM system has been assessed previously in AF and complex AT ablation procedures (8, 25, 27–29). In AF even two prospective, single-arm, international multi-center and nonrandomized clinical studies (UNCOVER-AF, NCT02825992; and RECOVER-AF, NCT03368781) have been conducted to address both procedural and long-term outcomes of CA procedures guided by CDM. Previous studies suggest that one of the most important advantages dipole charge density mapping offers is that variation in CL and CS activation changes do not influence the interpretation of the AT (7). In contrast with sequential 3D mapping systems, the CDM algorithm allows the mapping of multiple clusters of ATs and automatically distinguish the beats pertaining to the clinical AT; therefore, it did not hamper the accuracy of the activation maps.

### Recording technique

The applied recording technique (bipolar vs. unipolar) has considerable influence on the activation and voltage maps, thereby effecting the discrimination between different mechanisms but also selection of target sites for ablation (30). Signals mapped with the CDM system represent cardiac electrical charge (coulomb/cm^2^). As such, they are also subject to far field influence such as the QRS complex, even if to a lesser degree than voltage signals. This is simply because of the magnitude of the ventricles compared to the atria. In addition, the morphology of the unipolar signal has a lot of information, which is lost when subtraction is done to obtain bipolar voltage signals. In daily clinical practice, bipolar voltage signals are generally used as a surrogate for atrial substrate and mechanism analysis (focal: negative QS pattern, vs. reentrant: Positive-negative RS pattern), despite being influenced by wavefront propagation and CL. For example, perpendicular wavefront significantly decreases signal amplitude, and could therefore potentially overestimate the presence of low voltage during bipolar recordings (31, 32).

## Limitations

The retrospective nature of the present study and the limited number of included patients are the main limitations; however, we included a relatively high number of patients with AT undergoing CA after surgical interventions using the novel dipole charge density mapping system, considering that there were no previous studies of this kind. Further randomized multicenter studies need to be carried out in order to validate our findings. Patient selection for either CARTO or DCM mapping system was based on the operator's discretion. The authors recognize that this might introduce selection bias. Although not statistically significant, the CDM procedures took an average 40 min longer than CARTO procedures. This can be explained by the setup of the CDM system and the previously described steep learning curve this technology requires (9). As described in detail in Tables 1 and 3, congenital heart disease was more common in the CARTO group as compared to the CDM patient group. On the other hand, the CDM group consists of patients undergoing complex surgical procedures resulting in significant scarring of the atria. The authors recognize that the heterogeneity of the patient groups regarding previous procedure type might be a limitation. The authors acknowledge that whereas this study is a feasibility study, in future studies the feasibility of the utilized mapping system should be assessed in a broader context such as: cost-effectiveness and risk assessment.

## Conclusion

In conclusion, charge density based mapping might have the potential to provide improved long-term outcome once it is implemented in everyday clinical practice for CA of post-surgical ATs.

## Clinical perspectives

### Clinical competencies

In contrast with sequential 3D mapping systems, the novel charge density based mapping system (CDM) uses ultrasound to reconstruct and delineate the anatomy in detail, and combines real-time anatomy with consecutive activation history maps that can be performed on a beat-to-beat basis. One of the advantages CDM potentially offers is that variation in CL and CS activation changes do not influence the interpretation of the AT. In this study we found that CDM may potentially further improve long-term outcome once it is implemented in everyday clinical practice.

### Translational outlook

The implementation of CDM system can potentially transform the treatment of post-surgical AT by implementing to a more efficient approach. It can improve outcome especially in patients with multiple ATs and complex substrate. Quasi real time echocardiographic evaluation of the underlying anatomy will be a great asset to these difficult procedures.

## Data Availability

The raw data supporting the conclusions of this article will be made available by the authors, without undue reservation.
